# Monostable Dynamic Analysis of Microbeam-Based Resonators via an Improved One Degree of Freedom Model

**DOI:** 10.3390/mi9020089

**Published:** 2018-02-22

**Authors:** Lei Li, Qichang Zhang, Wei Wang, Jianxin Han

**Affiliations:** 1 School of Transportation and Vehicle Engineering, Shandong University of Technology, Zibo 255049, China; 2 Department of Mechanics, School of Mechanical Engineering, Tianjin University, Tianjin 300350, China; qzhang@tju.edu.cn (Q.Z.); wangweifrancis@tju.edu.cn (W.W.); 3 Tianjin Key Laboratory of High Speed Cutting and Precision Machining, Tianjin University of Technology and Education, Tianjin 300222, China; hanjianxin@tju.edu.cn

**Keywords:** MEMS, monostable vibration, Nonlinear Galerkin method, optimization

## Abstract

Monostable vibration can eliminate dynamic bifurcation and improve system stability, which is required in many microelectromechanical systems (MEMS) applications, such as microbeam-based and comb-driven resonators. This article aims to theoretically investigate the monostable vibration in size-effected MEMS via a low dimensional model. An improved single degree of freedom model to describe electrically actuated microbeam-based resonators is obtained by using modified couple stress theory and Nonlinear Galerkin method. Static displacement, pull-in voltage, resonant frequency and especially the monostable dynamic behaviors of the resonators are investigated in detail. Through perturbation analysis, an approximate average equation is derived by the application of the method of Multiple Scales. Theoretical expressions about parameter space and maximum amplitude of monostable vibration are then deduced. Results show that this improved model can describe the static behavior more accurately than that of single degree of freedom model via traditional Galerkin Method. This desired monostable large amplitude vibration is significantly affected by the ratio of the gap width to mircobeam thickness. The optimization design results show that reasonable decrease of this ratio can be beneficial to monostable vibration. All these analytical results are verified by numerical results via Differential Quadrature method, which show excellent agreement with each other. This analysis has the potential of improving dynamic performance in MEMS.

## 1. Introduction

Microbeam-based structures are widely applied in MEMS, such as microactuator/sensor [1,2,3], energy harvester [4], microresonator [5,6,7], gyroscope [8], microgripper [9,10] and so on. Their light weight, small size, low-energy consumption and durability make them even more attractive. MEMS pressure sensors and accelerometers are widely used in the automotive industry. Some microsensors and actuators are also adopted for various biomedical applications. In general, the operation of these electrostatically actuated resonant devices is based on linear resonance [11]. However, these dynamic systems are nonlinear and the output energy is very small in the case of linear resonance, which is undesirable in MEMS. Therefore, monostable large amplitude vibration is required in MEMS sensing application. It can eliminate dynamic bifurcation phenomenon and improve system stability. Zhang et al. [12] studied the dynamic behavior of comb-driven resonators with inclination of the fingers and edge effect on the capacitance and obtain parameters for linear resonance operation. The inclination of the fingers can be beneficial to restrain electrostatic nonlinearity and make the system realize linear vibration. Han et al. [13] studied dynamic behaviors of a doubly clamped microresonator and presented some design considerations to realize large amplitude vibration. Masri et al. [14] studied the stability of MEMS resonators when undergoing large amplitude motion with a delayed feedback velocity controller.

With the reduction of the scale of the MEMS, classic continuum mechanics theories are unable to describe the size effects [15], thus higher-order continuum theories or non-classic theories are inevitably needed to describe scale effect and corresponding relations in continuum mechanics [16]. The couple stress theory is considered as one of the higher-order continuum theories [17]. It involves parameters for expressing the effect of material length scale which possess the capability to describe the size effect of microstructures [18]. This theory includes two extra material length scale parameters besides the two classic material constants for elastic isotropic materials. Anthoine [19] and Yang et al. [20] presented the modified couple stress theory in which the two material length scale parameters are decreased to only one parameter. This feature facilitates the use of the modified couple stress theory for the study of micro- and nano-scale structures.

Static/dynamic behaviors of electrically actuated microbeams have been studied a lot by using different models and approaches. These investigations can be divided into two groups. The first group focuses on lumped-mass model and establishes single degree of freedom equation. Then, perturbation method is introduced to study dynamic behaviors of microbeam, which provides theoretical guidance for engineering [21,22,23,24]. However, lumped-mass model cannot accurately describe dynamic characteristics of mircobeam as the increase of amplitude. The other group focuses on continuum model and establishes partial differential equations which are then solved with a variety of numerical methods, such as Galerkin discretization and Differential Quadrature method. These results can describe dynamic characteristics of mircobeam more accurately [11,25,26,27]. Although the continuum model has high accuracy, it is not conducive to theoretical analysis in depth. Based on the continuum model, Younis et al. [28,29,30,31,32,33,34] studied static pull-in behavior and dynamic pull-in behavior of electrically actuated microbeams by using the Galerkin method, the Differential Quadrature method and the Shooting method. Results showed that the single degree of freedom model cannot accurately describe static displacement, pull-in voltage, resonant frequency or vibration amplitude. Specially, when amplitude exceeds half of the gap, the error between single degree of freedom model and continuum model increases significantly. Besides, Nayfeh et al. [23] analyzed the vibration behaviors of a Euler-Bernoulli beam with direct application of the method of multiple scales to the governing partial-differential equation. However, high-order vibration items were not considered in their study. In fact, high-order vibration items have important influence on dynamic behavior [25]. Fortunately, the Nonlinear Galerkin method can solve it with introducing higher modes into the single degree of freedom model [35].

The Nonlinear Galerkin method and the well-known Galerkin method can be used to obtain the low dimensional manifold by some projection onto a sub manifold [35,36]. However, the well-known Galerkin method restrict the sub-manifold at being a flat sub-manifold; the Nonlinear Galerkin method tries to improve on this by not restricting the sub-manifold to an affine sub-space. Kang et al. [37] studied dynamic behaviors of low-dimensional modeling of the fluid dynamic system with the Nonlinear Galerkin method. Considering the effect of higher modes, the Nonlinear Galerkin method can give an accurate description for the dynamic behaviors of the system. With the Nonlinear Galerkin method, partial differential equations can be discretized into a finite-degree-of-freedom system consisting of ordinary-differential equations and then, a low-dimensional model containing higher modes information can be generated.

It can be concluded from the above analysis that monostable vibration can eliminate dynamic bifurcation, improve system stability and increase the output of energy, which is desired in many MEMS applications. However, to the best of our knowledge, there are fewer effective methods to study monostable large amplitude vibration behaviors via the low dimensional model. This paper aims to obtain an improved single degree of freedom model by using modified couple stress theory and Nonlinear Galerkin method and deduce theoretical expressions about parameter space and maximum amplitude of monostable vibration.

The rest of this paper is organized as follows. In Section 2, a novel single degree of freedom model to describe electrically actuated microbeam-based resonators is obtained by using modified couple stress theory and Nonlinear Galerkin method. In Section 3, the method of Multiple Scales is used to derive an approximate average equation. In Section 4, static and dynamic properties of these devices are then investigated in detail. Parameter space and maximum amplitude of the monostable vibration are theoretically derived and numerically verified. Concluding remarks are given in the last section.

## 2. Mathematical Model

### 2.1. Governing Equation

**Figure 1:**
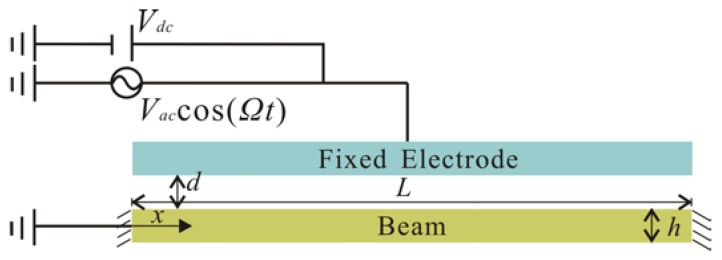
Schematic of an electrically actuated microbeam.

Here, we consider a clamped-clamped microbeam-based resonator, as shown in Figure 1. The actuation of the microbeam is realized by means of a bias voltage and an AC voltage component. The microbeam and the electrode are made from silicon material. Based on the modified couple stress theory, the equation of motion that governs the transverse deflection $\hat{w}(x,t)$ is written as [17].

(1) $$\rho A\ddot{\hat{w}}+(EI+\mu Al^{2}){\hat{w}}^{iv}+c\dot{\hat{w}}=(\hat{N}+\frac{EA}{2L}\int_{0}^{L}{{\hat{w}}^{\prime }}^{2}d\hat{x}){\hat{w}}^{\prime \prime }+\frac{\varepsilon_{0}b{\left[V_{dc}+V_{ac}\cos(\hat{\Omega }t)\right]}^{2}}{2{\left(d-\hat{w}\right)}^{2}}$$

with the following boundary conditions

(2) $$\hat{w}(0,\hat{t})={\hat{w}}^{\prime }(0,\hat{t})=\hat{w}(L,\hat{t})={\hat{w}}^{\prime }(L,\hat{t})=0$$

where $\dot{\hat{w}}=\frac{\partial \hat{w}}{\partial \hat{t}}$ and ${\hat{w}}^{\prime }=\frac{\partial \hat{w}}{\partial \hat{x}}$.

The first term on the right hand of Equation (1) represents the axial force and mid-plane stretching effects. Here, $\hat{x}$ is the position along the microbeam length; *A* and *I* are the area and moment of inertia of the cross section; *d* is the gap width; $\varepsilon_{0}$ is the dielectric constant of the gap medium. The parameter *N* corresponds to a tensile or compressive axial load, depending on whether it is positive or negative. *l* is introduced into Equation (1) as the material length scale parameter that has the capability to physically model properties of the couple stress effect. The last term in Equation (1) represents the parallel-plate electric actuation which is composed of DC and AC components. Here, DC voltage can cause a static deflection in the microbeam. There is a limit for the applied DC voltage called the static pull-in voltage [38]. AC voltage, which is small compared to DC voltage, causes the dynamic response of microbeam. Part of system parameters are defined as stated in Table 1.

In the relations above, *μ* is Lame’s constant that is defined by Young’s modulus *E* and Poisson’s ratio *ν* as

(3) $$\mu =\frac{E}{2(1+\upsilon )}$$

For convenience, the following non-dimensional variables are introduced

(4) $$w=\frac{\hat{w}}{d},\;x=\frac{\hat{x}}{L},\;\;t=\hat{t}\sqrt{\frac{EI}{\rho AL^{4}}}$$

Substituting the non-dimensional variables into Equations (1) and (2), yields the following non-dimensional equation of motion of the micro-resonator

(5) $$\ddot{w}+(1+\eta )w^{iv}+c_{n}\dot{w}-(N+\alpha_{1}\int_{0}^{1}{w^{\prime }}^{2}dx)w^{\prime \prime }=\alpha_{2}\frac{{\left(V_{dc}+V_{ac}\cos\Omega t\right)}^{2}}{{\left(1-w\right)}^{2}}$$

with boundary conditions

(6) $$w(0,t)=w^{\prime }(0,t)=w(1,t)=w^{\prime }(1,t)=0$$

The parameters appearing in Equation (5) are

(7) $$\alpha_{1}=6\times {\left(\frac{d}{h}\right)}^{2},\;\alpha_{2}=\frac{6\varepsilon_{0}L^{4}}{Ed^{3}h^{3}},\;\eta =\frac{\mu Al^{2}}{EI},\;N=\frac{\hat{N}L^{2}}{EI}$$

where $\alpha_{1}$ represents ratio coefficient of the gap width to the mircobeam thickness, $\alpha_{2}$ represents electrostatic force coefficient and η represents scale effect.

### 2.2. The Nonlinear Galerkin Method

Compared with the Linear Galerkin method, the Nonlinear Galerkin method can describe the dynamic behaviors of the system more accurately. In this section, we introduce the Nonlinear Galerkin method to deal with Equation (5) and obtain an improved one degree of freedom model [36].

Firstly, considering the Galerkin procedure, we discretize Equation (5) into a finite-degree-of-freedom system consisting of ordinary differential equations. This technique and its application to nonlinear systems were discussed by Abdel-Rahman et al. [39].

The solution of Equation (5) can be expressed as

(8) $$w(x,t)=\sum_{i=1}^{\infty }u_{i}(t)\phi_{i}(x)$$

where $\phi_{i}$ is the *i*-th linear undamped mode shape of the straight microbeam, normalized such that $\int_{0}^{1}\phi_{i}\phi_{j}dx=\delta_{ij}$ and governed by

(9) $$(1+\eta )\phi_{i}^{iv}=N\phi_{i}{}^{\prime \prime }+\omega_{i}^{2}\phi_{i}$$

with

(10) $$\phi_{i}(0)=\phi_{i}(1)=\phi_{i}{}^{\prime }(0)=\phi_{i}{}^{\prime }(1)=0$$

where $\omega_{i}$ is the *i*-th natural frequency of the microbeam. Equations (9) and (10) represent a boundary-value problem that can be solved by using a combination of Shooting method and a bisection procedure for each pair of mode shape and natural frequency [39].

Then, Equation (5) is multiplied by ${\left(1-w\right)}^{2}$ [25], so that the electric-force term is represented exactly. Substituting Equation (8) into the resulting equation, multiplying by $\phi_{i}$ and integrating the outcome from *x* = 0–1, yield

(11) $$\begin{array}{l} {\ddot{u}}_{n}+\omega_{n}^{2}u_{n}+c_{n}{\dot{u}}_{n}-2\sum_{i,j=1}^{M}u_{i}{\ddot{u}}_{j}\int_{0}^{1}\phi_{i}\phi_{j}\phi_{n}dx+\sum_{i,j,k=1}^{M}u_{i}u_{j}{\ddot{u}}_{k}\int_{0}^{1}\phi_{i}\phi_{j}\phi_{k}\phi_{n}dx\\ =2\sum_{i,j=1}^{M}\omega_{i}^{2}u_{i}u_{j}\int_{0}^{1}\phi_{i}\phi_{j}\phi_{n}dx-\sum_{i,j,k=1}^{M}\omega_{i}^{2}u_{i}u_{j}u_{k}\int_{0}^{1}\phi_{i}\phi_{j}\phi_{k}\phi_{n}dx+2c_{n}\sum_{i,j=1}^{M}u_{i}{\dot{u}}_{j}\int_{0}^{1}\phi_{i}\phi_{j}\phi_{n}dx\\ -c_{n}\sum_{i,j,k=1}^{M}u_{i}u_{j}{\dot{u}}_{k}\int_{0}^{1}\phi_{i}\phi_{j}\phi_{k}\phi_{n}dx+\alpha_{1}\sum_{i,j,k=1}^{M}u_{i}u_{j}u_{k}\Gamma (\phi_{i},\phi_{j})\int_{0}^{1}\phi_{k}^{\prime \prime }\phi_{n}dx\\ -2\alpha_{1}\sum_{i,j,k,l=1}^{M}u_{i}u_{j}u_{k}u_{l}\Gamma (\phi_{i},\phi_{j})\int_{0}^{1}\phi_{k}\phi_{l}^{\prime \prime }\phi_{n}dx+\alpha_{1}\sum_{i,j,k,l,m=1}^{M}u_{i}u_{j}u_{k}u_{l}u_{m}\Gamma (\phi_{i},\phi_{j})\int_{0}^{1}\phi_{k}\phi_{l}\phi_{m}^{\prime \prime }\phi_{n}dx\\ +\alpha_{2}{\left(V_{dc}+V_{ac}\cos\Omega t\right)}^{2}\int_{0}^{1}\phi_{n}dx \end{array}$$

where $\Gamma (\phi_{i},\phi_{j})=\int_{0}^{1}\phi_{i}\phi_{j}dx$. Due to $V_{dc}>>V_{ac}$ [34], ${\left(V_{dc}+V_{ac}\cos\Omega t\right)}^{2}\approx V_{dc}^{2}+2V_{dc}V_{ac}\cos\Omega t$ is obtained.

Equation (11) represents a discretized system consisting of ordinary-differential equations, which contain all nonlinearities up to fifth order. The above is the Linear Galerkin method for dimensionality reduction. Most researchers utilized the derived reduced-order models to simulate the static behavior and dynamic response of microbeam-based MEMS devices. However, the way is not conducive to theoretical analysis. Here, the Nonlinear Galerkin method is introduced to deal with the above equations.

Starting with an abstract setting, a nonlinear dynamical system is separated into the linear and the higher order nonlinear part

(12) $$\dot{x}+g(x,t)=\dot{x}+Ax+h(x,t)=0,x\in R^{2\times M}$$

with Ax as linear part and h(x,t) as nonlinear part of the system g(x,t). Here we take 2*M* spatial dimension corresponding with Equation (11).

Here, we assume the solution x as

(13) $$x=Y_{m}\xi +Z_{m}\eta$$

where the columns of the matrix $Y_{m}=[y_{1},\cdots ,y_{m}]$ span the m-dimensional sub-space span $\{y_{1},\cdots ,y_{m}\}$ and the columns of the matrix $Z_{m}=[y_{m+1},\cdots ,y_{2\times M}]$ span complementary sub-space span $\{y_{m+1},\cdots ,y_{2\times M}\}$.

Substituting Equation (13) into Equation (12), multiplying by ${\tilde{Y}}_{m}^{T}$ and ${\tilde{Z}}_{m}^{T}$ from the left, yield

(14) $$\begin{array}{l} \dot{\xi }+{\tilde{Y}}^{T}AY\xi +{\tilde{Y}}^{T}h(Y\xi +Z\eta ,t)=0\\ \dot{\eta }+{\tilde{Z}}^{T}AZ\eta +{\tilde{Z}}^{T}h(Y\xi +Z\eta ,t)=0 \end{array}$$

where ${\tilde{Y}}_{m}^{T}Y_{m}=I,\;{\tilde{Z}}_{m}^{T}Z_{m}=I$ and I is unit matrix.

Combination with the dynamic response of microbeam, ξ represents low-frequency part and η represents high-frequency part. To describe dynamic response of system accurately with the low-frequency part, the following relation is introduced

(15) η=ϕ(ξ)

Substituting Equation (15) into Equation (14), we can obtain

(16) $$\dot{\xi }+{\tilde{Y}}^{T}AY\xi +{\tilde{Y}}^{T}h(Y\xi +Z\phi (\xi ),t)=0$$

Both low-frequency part and high-frequency part are introduced in Equation (16). It is considered that the high-frequency vibration has little impact on system dynamics under primary resonance condition. So, we set $\dot{\eta }=0$. Then, we can obtain the reduced order model that contains the information of high order modes.

To obtain dynamic equation of single degree of freedom, we set subspace dimension equal to 2.

In order to simplify the calculation, we set *M* equal to 3 and only keep linear part of the high dimensional space variables. The relationship between the high-frequency part and low-frequency part is obtained, as shown below

(17) $$\begin{array}{l} u_{3}=\frac{1}{\omega_{3}^{2}}[2\int_{0}^{1}\phi_{1}^{2}\phi_{3}dx(u_{1}{\ddot{u}}_{1}+c_{n}u_{1}{\dot{u}}_{1}+\omega_{1}^{2}u_{1}^{2})-u_{1}^{2}{\ddot{u}}_{1}\int_{0}^{1}\phi_{1}^{3}\phi_{3}dx-\omega_{1}^{2}u_{1}^{3}\int_{0}^{1}\phi_{1}^{3}\phi_{3}dx-c_{n}u_{1}^{2}{\dot{u}}_{1}\int_{0}^{1}\phi_{1}^{3}\phi_{3}dx\\ \;+\alpha_{1}u_{1}^{3}\int_{0}^{1}{\phi_{1}^{\prime }}^{2}dx\int_{0}^{1}\phi_{1}{}^{\prime \prime }\phi_{3}dx-2\alpha_{1}u_{1}^{4}\int_{0}^{1}{\phi_{1}^{\prime }}^{2}dx\int_{0}^{1}\phi_{1}{}^{\prime \prime }\phi_{3}\phi_{1}dx+\alpha_{2}V_{dc}\int_{0}^{1}\phi_{3}dx] \end{array}$$

Substituting Equation (17) into Equation (11) and keeping all nonlinearities up to fifth order, yield the following novel single degree of freedom equation

(18) $$\begin{array}{l} \left({\ddot{u}}_{1}+\omega_{1}^{2}u_{1}+c_{n}{\dot{u}}_{1})(n_{0}+n_{1}u_{1}+n_{2}u_{1}^{2}+n_{3}u_{1}^{3}+n_{4}u_{1}^{4}\right)\\ =m_{1}u_{1}^{3}+m_{2}(u_{1}^{2}{\ddot{u}}_{1}+c_{n}u_{1}^{2}{\dot{u}}_{1})+m_{3}u_{1}+m_{4}+2m_{5}V_{dc}V_{ac}\cos\Omega t\\ \;+m_{6}u_{1}^{2}+m_{7}u_{1}^{4}+m_{8}(u_{1}^{3}{\ddot{u}}_{1}+c_{n}u_{1}^{3}{\dot{u}}_{1})+m_{9}u_{1}^{5} \end{array}$$

where coefficients are expressed as Equations (A1)–(A15) in Appendix A.

The above is the Nonlinear Galerkin method for dimensionality reduction. The previous single degree of freedom model obtained by the Linear Galerkin method only contains the first mode. In this paper, the Nonlinear Galerkin method is introduced to obtain the improved single degree of freedom model that contains the first mode and the third mode, which improves the accuracy of the model.

This paper aims to study monostable large amplitude vibration. With the increase of amplitude, the advantage of the improved model becomes more and more obvious.

## 3. Perturbation Analysis

Static analysis is significant in the MEMS. Through it, equilibrium position, pull-in voltage and pull-in location of the system can be obtained. Each of the DC voltage corresponds to a static displacement and the perturbation method is used to obtain an approximate solution near the equilibrium position. We assume $u_{1}=u_{1d}+u_{1s}$, where $u_{1d}$ and $u_{1s}$ represent dynamic behavior and static behavior, respectively. Then, we can obtain the static equation by making the $u_{1}$ independent of time

(19) $$\omega_{1}^{2}u_{1s}(n_{0}+n_{1}u_{1s}+n_{2}u_{1s}^{2}+n_{3}u_{1s}^{3}+n_{4}u_{1s}^{4})=m_{1}u_{1s}^{3}+m_{3}u_{1s}+m_{4}+m_{6}u_{1s}^{2}+m_{7}u_{1s}^{4}+m_{9}u_{1s}^{5}$$

and the dynamic equation by ignoring high order damping terms

(20) $$\begin{array}{ll} {\ddot{u}}_{1d}+\omega_{1d}^{2}u_{1d}+c_{n}{\dot{u}}_{1d}= & q_{1}u_{1d}{\ddot{u}}_{1d}+q_{2}u_{1d}^{2}{\ddot{u}}_{1d}+q_{3}u_{1d}^{3}{\ddot{u}}_{1d}+q_{4}u_{1d}^{4}{\ddot{u}}_{1d}+q_{5}u_{1d}^{2}\\ & +q_{6}u_{1d}^{3}+q_{7}u_{1d}^{4}+q_{8}u_{1d}^{5}+q_{9}V_{ac}\cos\Omega t \end{array}$$

where coefficients are expressed as Equations (A16)–(A25) in Appendix A.

To indicate the significance of each term in the equation of motion, ε is introduced as a small non-dimensional bookkeeping parameter. Considering the electrostatic force term $q_{9}=O(\varepsilon^{5})$, scaling the dissipative terms, we obtain

(21) $$\begin{array}{ll} {\ddot{u}}_{1d}+\omega_{1d}^{2}u_{1d}+\varepsilon^{4}c_{n}{\dot{u}}_{1d} & =q_{1}u_{1d}{\ddot{u}}_{1d}+q_{2}u_{1d}^{2}{\ddot{u}}_{1d}+q_{3}u_{1d}^{3}{\ddot{u}}_{1d}+q_{4}u_{1d}^{4}{\ddot{u}}_{1d}+q_{5}u_{1d}^{2}\\ & +q_{6}u_{1d}^{3}+q_{7}u_{1d}^{4}+q_{8}u_{1d}^{5}+\varepsilon^{5}q_{9}V_{ac}\cos\Omega t \end{array}$$

To express the relationship between the excitation frequency and the natural frequency, we introduce a detuning parameter σ defined by $\Omega =\omega_{1d}+\varepsilon^{4}\sigma$. Here, σ is the tuning parameter. Then, to determine a fifth-order uniform expansion of the solution of Equation (21) by using the method of Multiple Scales, we introduce three time scales $T_{0}=t$, $T_{2}=\varepsilon^{2}t$ and $T_{4}=\varepsilon^{4}t$ [23] and expand the time-dependent variable $u_{1d}$ in powers of ε as

(22) $$u_{1d}=\varepsilon v_{1}(T_{0},T_{2},T_{4})+\varepsilon^{2}v_{2}(T_{0},T_{2},T_{4})+\varepsilon^{3}v_{3}(T_{0},T_{2},T_{4})+\varepsilon^{4}v_{4}(T_{0},T_{2},T_{4})+\varepsilon^{5}v_{5}(T_{0},T_{2},T_{4})$$

Substituting Equation (22) into Equation (21) and equating coefficients of like powers of ε, yields

order ε:

(23) $$\frac{\partial^{2}v_{1}}{\partial T_{0}^{2}}+\omega_{1d}^{2}v_{1}=0$$

order $\varepsilon^{2}$:

(24) $$\frac{\partial^{2}v_{2}}{\partial T_{0}^{2}}+\omega_{1d}^{2}v_{2}=q_{1}v_{1}\frac{\partial^{2}v_{1}}{\partial T_{0}^{2}}+q_{5}v_{1}^{2}$$

order $\varepsilon^{3}$:

(25) $$\frac{\partial^{2}v_{3}}{\partial T_{0}^{2}}+\omega_{1d}^{2}v_{3}=-2\frac{\partial^{2}v_{1}}{\partial T_{0}\partial T_{2}}+q_{1}v_{2}\frac{\partial^{2}v_{1}}{\partial T_{0}^{2}}+q_{1}v_{1}\frac{\partial^{2}v_{2}}{\partial T_{0}^{2}}+2q_{5}v_{2}v_{1}+q_{2}v_{1}^{2}\frac{\partial^{2}v_{1}}{\partial T_{0}^{2}}+q_{6}v_{1}^{3}$$

order $\varepsilon^{4}$:

(26) $$\begin{array}{ll} \frac{\partial^{2}v_{4}}{\partial T_{0}^{2}}+\omega_{1d}^{2}v_{4} & =-2D_{0}D_{2}v_{2}+q_{1}(v_{1}\frac{\partial^{2}v_{3}}{\partial T_{0}^{2}}+v_{3}\frac{\partial^{2}v_{1}}{\partial T_{0}^{2}}+v_{2}\frac{\partial^{2}v_{2}}{\partial T_{0}^{2}}+2v_{1}\frac{\partial^{2}v_{1}}{\partial T_{0}\partial T_{2}})\\ & +q_{2}(2v_{2}v_{1}\frac{\partial^{2}v_{1}}{\partial T_{0}^{2}}+v_{1}^{2}\frac{\partial^{2}v_{2}}{\partial T_{0}^{2}})+q_{3}v_{1}^{3}\frac{\partial^{2}v_{1}}{\partial T_{0}^{2}}+q_{5}(2v_{1}v_{3}+v_{2}^{2})+3q_{6}v_{1}^{2}v_{2}+q_{7}v_{1}^{4} \end{array}$$

order $\varepsilon^{5}$:

(27) $$\begin{array}{ll} \frac{\partial^{2}v_{5}}{\partial T_{0}^{2}}+\omega_{1d}^{2}v_{5} & =-2D_{0}D_{4}v_{1}-2D_{0}D_{2}v_{3}-D_{2}D_{2}v_{1}-c_{n}\frac{\partial v_{1}}{\partial T_{0}}+q_{1}(v_{1}\frac{\partial^{2}v_{4}}{\partial T_{0}^{2}}+v_{4}\frac{\partial^{2}v_{1}}{\partial T_{0}^{2}}\\ & +v_{3}\frac{\partial^{2}v_{2}}{\partial T_{0}^{2}}+v_{2}\frac{\partial^{2}v_{3}}{\partial T_{0}^{2}}+2v_{1}\frac{\partial^{2}v_{2}}{\partial T_{0}\partial T_{2}}+2v_{2}\frac{\partial^{2}v_{1}}{\partial T_{0}\partial T_{2}})+4q_{7}v_{1}^{3}v_{2}+q_{8}v_{1}^{5}+q_{2}(2v_{3}v_{1}\frac{\partial^{2}v_{1}}{\partial T_{0}^{2}}\\ & +v_{1}^{2}\frac{\partial^{2}v_{3}}{\partial T_{0}^{2}}+2v_{2}v_{1}\frac{\partial^{2}v_{2}}{\partial T_{0}^{2}}+v_{2}^{2}\frac{\partial^{2}v_{1}}{\partial T_{0}^{2}})+q_{3}(v_{1}^{3}\frac{\partial^{2}v_{2}}{\partial T_{0}^{2}}+3v_{2}v_{1}^{2}\frac{\partial^{2}v_{1}}{\partial T_{0}^{2}})+q_{4}v_{1}^{4}\frac{\partial^{2}v_{1}}{\partial T_{0}^{2}}\\ & +q_{5}(2v_{1}v_{4}+2v_{2}v_{3})+3q_{6}(v_{2}^{2}v_{1}+v_{1}^{2}v_{3})+q_{9}V_{ac}\cos\Omega t \end{array}$$

where $D_{n}=\partial /\partial T_{n}$.

The solution of Equation (23) can be expressed as

(28) $$v_{1}=A(T_{2},T_{4})\exp(i\omega_{1d}T_{0})+\bar{A}(T_{2},T_{4})\exp(-i\omega_{1d}T_{0})$$

where the overbar indicates the complex conjugate. The function $A(T_{2},T_{4})$ can be determined by eliminating the secular terms from Equations (24)–(27).

Expressing the amplitude *A* in the polar form $A(T_{2},T_{4})=\frac{1}{2}ae^{i\theta }$, where a and θ are real functions of $T_{2}$ and $T_{4}$ and separating secular terms into its real and imaginary parts, we can obtain the following average equation

(29) $$a^{\prime }=-\frac{1}{2}c_{n}a+\frac{1}{2}\frac{q_{9}V_{ac}}{\omega_{1d}}\sin\varphi$$

(30) $$a\varphi^{\prime }=\sigma a+\frac{1}{8}\frac{\kappa_{1}a^{3}}{\omega_{1d}}+\frac{1}{32}\frac{\kappa_{2}a^{5}}{\omega_{1d}}+\frac{1}{2}\frac{q_{9}V_{ac}}{\omega_{1d}}\cos\varphi$$

where $\varphi =\sigma T_{4}-\theta$, $a^{\prime }=\partial a/\partial T_{2}+\partial a/\partial T_{4}$, $\varphi^{\prime }=\partial \varphi /\partial T_{2}+\partial \varphi /\partial T_{4}$, $\kappa_{1}=(q_{5}-q_{1}\omega_{1d}^{2})(\frac{10q_{5}}{3\omega_{1d}^{2}}-\frac{1}{3}q_{1})+3(q_{6}-q_{2}\omega_{1d}^{2})$ and $\kappa_{2}$ is expressed as Equations (A26)–(A29) in Appendix A.

Then, the steady-state frequency response can be obtained by solving the following frequency response equation

(31) $$[{\left(\frac{1}{2}c_{n}\right)}^{2}+{\left(\sigma +\frac{1}{8}\frac{\kappa_{1}a^{2}}{\omega_{1d}}+\frac{1}{32}\frac{\kappa_{2}a^{4}}{\omega_{1d}}\right)}^{2}]a^{2}=\frac{q_{9}^{2}V_{ac}^{2}}{4\omega_{1d}^{2}}$$

The relationship between the resonance frequency shift and the maximum amplitude of oscillation is derived as

(32) $$\sigma =-\frac{1}{8}\frac{\kappa_{1}a_{\max}^{2}}{\omega_{1d}}-\frac{1}{32}\frac{\kappa_{2}a_{\max}^{4}}{\omega_{1d}}$$

where $a_{\max}=q_{9}V_{ac}/\omega_{1d}c_{n}$.

By inspection, when the $\kappa_{1}a^{2}/8+\kappa_{2}a^{4}/32$ monotonically changes with the increase of amplitude, it is clear that the device will experience hardening if $\kappa_{1}a^{2}/8+\kappa_{2}a^{4}/32<0$ and will experience softening if $\kappa_{1}a^{2}/8+\kappa_{2}a^{4}/32>0$. If the change of the value of $\kappa_{1}a^{2}/8+\kappa_{2}a^{4}/32$ is not monotonous, the system will experience hardening and softening simultaneously. For small amplitude oscillation, the hardening and softening of the system are decided by $\kappa_{1}$. However, with the increase of amplitude, the influence of high-order nonlinear terms on the system becomes more and more important. So, hardening and softening properties become very complex under the large amplitude vibration. Zhang et al. [22] and Nayfeh et al. [23] studied spring softening and hardening based on the traditional single degree of freedom model. However, with the increase of amplitude, it cannot describe dynamic behaviors of the system. Here, the monostable large amplitude vibration is studied with the improved single degree of freedom model. Following, taking *σ* as the bifurcation parameter and taking $V_{dc}$ and $V_{ac}$ as the unfolding parameters, we calculate unfolding of Equation (31). The traditional calculation method of the transition set of the system will lead to nonlinear equations containing high-order terms. Here, we only need to obtain parameter space of the monostable vibration. So, a new way is given.

The amplitude frequency curve can be decomposed into two parts, as the following

(33) $$\sigma =f_{1}(a)=-\frac{1}{8}\frac{\kappa_{1}a^{2}}{\omega_{1d}}-\frac{1}{32}\frac{\kappa_{2}a^{4}}{\omega_{1d}}+\sqrt{\frac{q_{9}^{2}V_{ac}^{2}}{4\omega_{1d}^{2}a^{2}}-{\left(\frac{1}{2}c_{n}\right)}^{2}}$$

(34) $$\sigma =f_{2}(a)=-\frac{1}{8}\frac{\kappa_{1}a^{2}}{\omega_{1d}}-\frac{1}{32}\frac{\kappa_{2}a^{4}}{\omega_{1d}}-\sqrt{\frac{q_{9}^{2}V_{ac}^{2}}{4\omega_{1d}^{2}a^{2}}-{\left(\frac{1}{2}c_{n}\right)}^{2}}$$

The monostable vibration appears when the frequency σ of the left amplitude frequency curve $f_{2}$ monotonically increases with the increase of amplitude and the frequency σ of the right amplitude frequency curve $f_{1}$ monotonically decreases with the increase of amplitude. If the curve $f_{1}$ monotonically decreases and the curve $f_{2}$ cannot monotonically change, the softening appears. On the contrary, if the curve $f_{2}$ monotonically increases and the curve $f_{1}$ cannot monotonically change, the hardening appears. If both the curve $f_{2}$ and the curve $f_{1}$ cannot monotonically change, the system will experience hardening and softening simultaneously. Here, the dynamic curve is in contact with mathematical function, which simplifies the calculation. Now, the necessary and sufficient conditions of the existence of the monostable vibration are obtained.

(35) $$\frac{df_{1}(a)}{da}<0\;\mathrm{and}\;\frac{df_{2}(a)}{da}>0\;\mathrm{for}\;a\in [0,a_{\max}]$$

## 4. Results and Discussion

In this section, Differential Quadrature method and Finite Element method are introduced to verify the accuracy of the model. Meanwhile, with unfolding analysis and optimization theory, parameter space and maximum amplitude of the monostable vibration are obtained.

### 4.1. Convergence Analysis

Convergence is a key problem. With the traditional single degree of freedom model, static displacement curve cannot be obtained accurately. To solve the problem, higher dimensional model was introduced in previous studies [39]. Here, the novel single degree of freedom model is used to handle this convergence problem. To validate accuracy of our model, finite element results are obtained from the Multiphysics simulation software. Meanwhile, compared with single degree of freedom model results obtained by using Linear Galerkin method, the ascendency of the Nonlinear Galerkin method appears. Here, we consider a microbeam without scale effect and axial stress.

**Figure 2:**
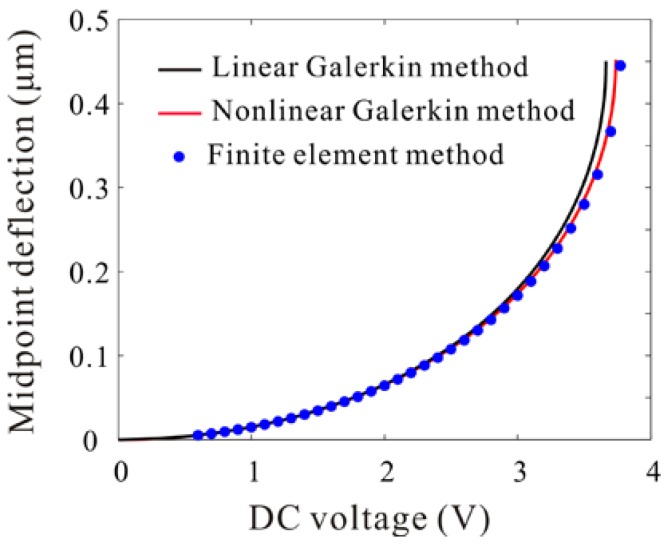
Comparison of the calculated midpoint deflection using nonlinear Galerkin method, linear Galerkin method and finite element method for various values of DC voltages under d=1 μm.

In Figure 2, the calculated static deflections of the microbeam obtained by using the Nonlinear Galerkin method are compared with those obtained by using Linear Galerkin method and Finite Element method. Results are presented from 0 V to pull-in voltage. It is noted from Figure 2 that the pull-in voltage predicted by the Nonlinear Galerkin method is more accurate than that predicted by Linear Galerkin method. The midpoint deflections predicted by those three methods are very close away from pull-in voltage but the midpoint deflections predicted by Linear Galerkin method deviate increasingly as pull-in is approached. The ascendency of the Nonlinear Galerkin method appears. Then, natural frequency under different DC voltage is obtained by using those three methods. As shown in Table 2, the error of results obtained by the Nonlinear Galerkin method is less than that obtained by the Linear Galerkin method. As pull-in is approached, the error of results obtained by Linear Galerkin method reaches to 9.0%. However, the error of results obtained by the Nonlinear Galerkin method is about 2.3%. When DC voltage is away from pull-in. the error caused by the Nonlinear Galerkin method is less than 1%. It is worth noting that the error is obtained by comparing the result of the theory and that of the simulation.

Besides, pull-in voltage is obtained by using those three methods under different gap width. As shown in Table 3, it can be seen that the result obtained by the Nonlinear Galerkin method agrees well with that obtained by Finite Element method, which demonstrates that the present analytical method is effective. However, the error of results obtained by Linear Galerkin method reaches to 7.1%.

Thus, our model is superior to the traditional single degree of freedom model. It can predict static displacement, natural frequency and pull-in voltage more accurately and convergence problem is also solved by our model.

### 4.2. Static Analysis

**Figure 3:**
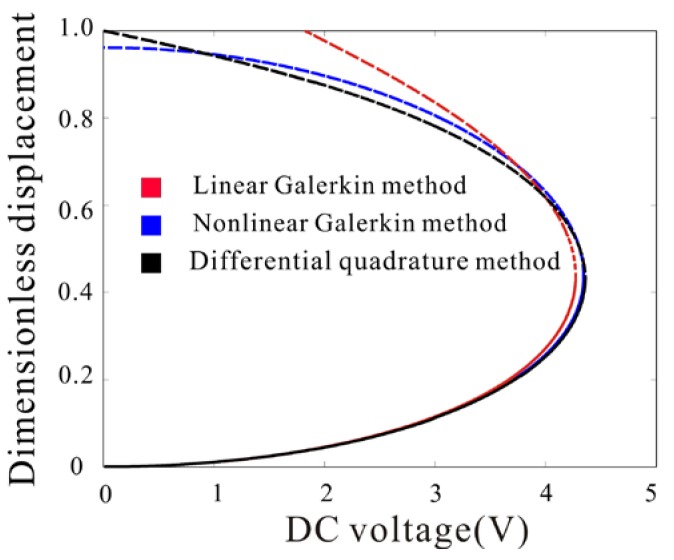
Comparison of the calculated maximum non-dimensional deflection using Nonlinear Galerkin method, Differential Quadrature method and linear Galerkin method for various values of DC voltages (Solid line: stable; dashed line: unstable).

In this section, scale effect and axial stress are considered. The static deflection and static pull-in voltage of microbeam are calculated with our model. Meanwhile, Differential Quadrature method is introduced to handle Equation (5) for numerical verification. The calculated static deflections of the microbeam with η=0.25, d=1 μm and subject to a stretched axial stress N=6 are shown in Figure 3. It can be noted from this figure that the pull-in voltage predicted by the Nonlinear Galerkin method is accurate while that predicted by the Linear Galerkin method has significant error. The lower branches predicted by those three methods are very close away from pull-in voltage but the branch predicted by the Linear Galerkin method deviates increasingly as pull-in is approached. Generally, the results obtained by using the Nonlinear Galerkin method are in excellent agreement with those obtained with the Differential Quadrature method. However, the static deflections of microbeam obtained by using the Linear Galerkin method are in poor agreement with them. Specially, when DC voltage approaches zero, the upper branch predicted by Linear Galerkin method is non-convergent. The upper branch represents potential barrier of the system. When the vibration amplitude approaches potential barrier, the results predicted by Linear Galerkin method have serious errors. With the Nonlinear Galerkin method, the misconvergence of potential barrier is solved.

What’s more, the calculation formula of the pull-in voltage can be obtained easily.

From Equation (19), the relationship between static displacement $u_{1s}$ and DC voltage $V_{dc}$ is obtained

(36) $$u_{1s}=f(V_{dc})$$

**Figure 4:**
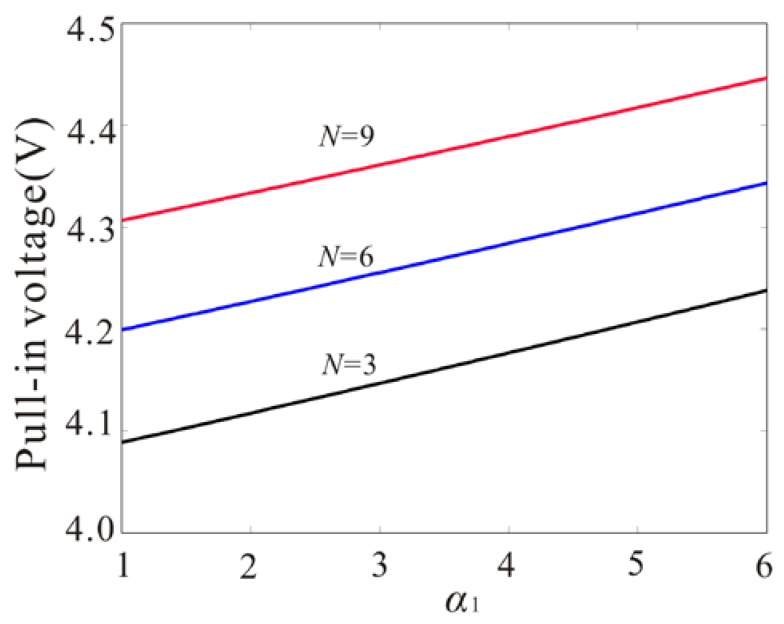
The influence of ratio of the gap width to mircobeam thickness and axial stress on the pull-in voltage.

When pull-in occurs, both branches collide and destroy each other with one eigenvalue tending to zero. Thus, the pull-in voltage corresponds to a saddle-node bifurcation. So, the pull-in occurs when $dV/du_{1s}=0$. As shown in Figure 4, the pull-in voltages under different size parameters and axial stress are given. To describe qualitatively the change of pull-in voltages, the electrostatic force coefficient $\alpha_{2}$ should remain constant with the increase of the ratio coefficient $\alpha_{1}$. It is noted from Figure 4 that the pull-in voltage increases with the increase of the ratio coefficient of the gap width to the mircobeam thickness. Meanwhile, a stretched axial stress can increase pull-in voltage. Driven by the same DC voltage, increasing the ratio of the gap width to the mircobeam thickness and positive axial stress is useful to prevent pull-in.

### 4.3. Dynamic Analysis

The monostable vibration is desired for many applications, such as microbeam resonator [40]. Here, parameter space and maximum amplitude about the monostable vibration are obtained.

#### 4.3.1. Small Vibration

**Figure 5:**
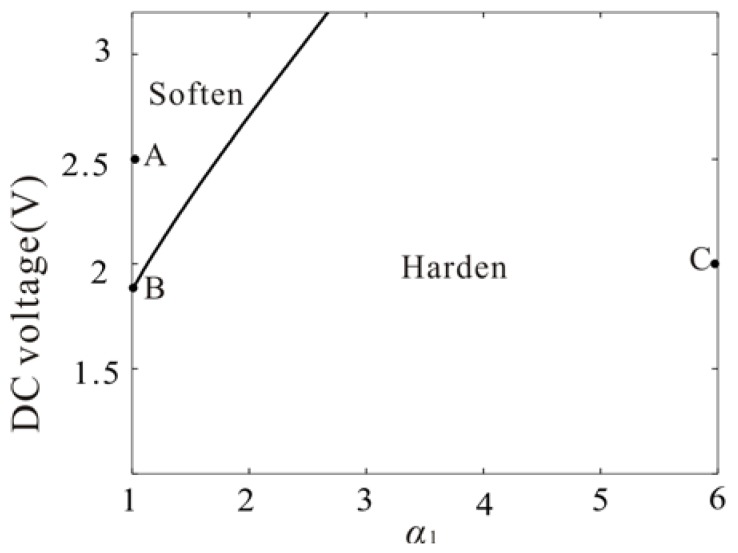
The parameter space of softening and hardening under the small vibration amplitudes.

In Figure 5, we study the influence of the ratio of the gap width to the mircobeam thickness and DC voltage on the hardening and softening properties of the system under the small amplitude oscillation. It is noted that the increase of the DC voltage and the decrease of the gap width can lead to softening phenomenon with $\kappa_{1}>0$. On the contrary, the decrease of the voltage and the increase of the gap width can lead to hardening phenomenon with $\kappa_{1}<0$. It is found that the mechanical spring is responsible for the hardening behavior and the electrostatic force is responsible for the softening behavior. From Figure 5, the curve represents the boundary between the softening area and hardening area. Near the boundary, there is no softening phenomenon or hardening phenomenon and the system will experience monostable vibration.

**Figure 6:**
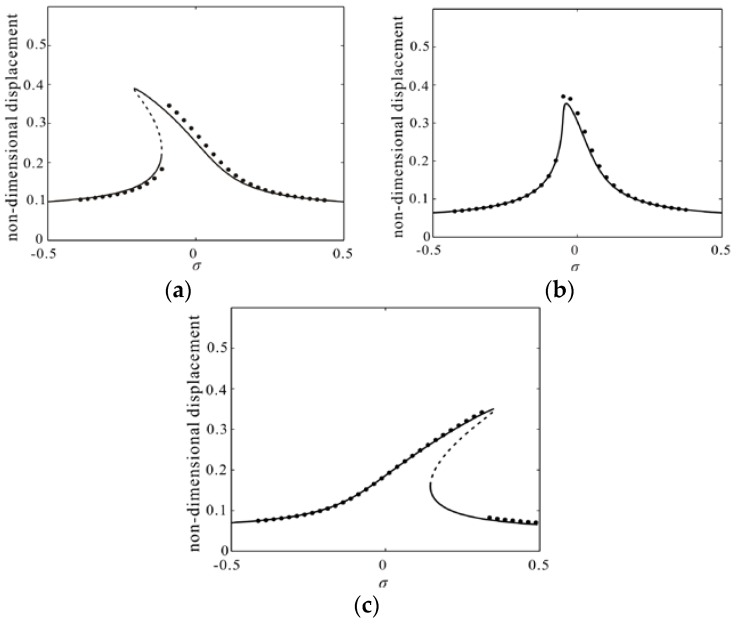
(**a**–**c**) Comparison of the frequency response curve obtained by nonlinear Galerkin method (line) and differential quadrature method (dotted line) corresponding to A–C in Figure 5 (solid line: stable; dashed line: unstable).

Three types of parameters (point A, point B and point C) are taken from softening area, hardening area and boundary as shown in Figure 5. And the amplitude frequency response curves of them are given as shown in Figure 6.

Figure 6a shows a representative frequency response to our problem when $\kappa_{1}>0$ in the case of $V_{dc}=2.5,\;V_{ac}=0.015,\;\alpha_{1}=1$. Here, appropriate excitation voltages are needed to introduce softening nonlinearity and prevent pull-in. And their stability is studied by using Routh Criterion. Figure 6b shows monostable vibration when $\kappa_{1}=0$ in the case of $V_{dc}=1.86,\;V_{ac}=0.022,\;\alpha_{1}=1$. At this time, the DC voltage and gap width should satisfy certain relations as shown in Figure 5. Figure 6c shows hardening nonlinearity when *κ*_1_ < 0 in the case of $V_{dc}=2,\;V_{ac}=0.02,\;\alpha_{1}=6$. Meanwhile, the results obtained by the Nonlinear Galerkin method are compared with them obtained by Differential Quadrature method. Here, we apply the way of frequency sweep into the Differential Quadrature method and they are in excellent agreement with each other.

#### 4.3.2. Monostable Large Amplitude Vibration

Due to the existence of nonlinear electrostatic force and geometric nonlinearity, it is hard to realize the monostable large amplitude vibration. The traditional single degree of freedom model is not enough to characterize the frequency response under the large amplitude vibration. In this section, we try to qualitatively study the monostable large amplitude vibration with the improved single degree of freedom model. To verify the validity of the results, the Differential Quadrature method is used to compare with the Nonlinear Galerkin method.

Section 4.3.1 shows that the nonlinear electrostatic force will make the device experience softening and the geometric nonlinearity will make device experience hardening. Under the small amplitude oscillation, to realize the monostable vibration, parameters should be selected near the boundary in Figure 5. And, from Equation (5), it is found that the growth rate of nonlinear electrostatic force is faster than that of geometric nonlinearity with the increase of amplitude of oscillation. The softening becomes dominant when the gap width decreases. So, under the large amplitude oscillation, the device tends to experience softening. In order to realize the monostable large amplitude vibration, we need select parameters near the boundary in Figure 5 and control softening with the increase of amplitude. Besides, as the AC voltage increases, the amplitude increases. More solutions will appear only when amplitude increases to a critical value.

**Figure 7:**
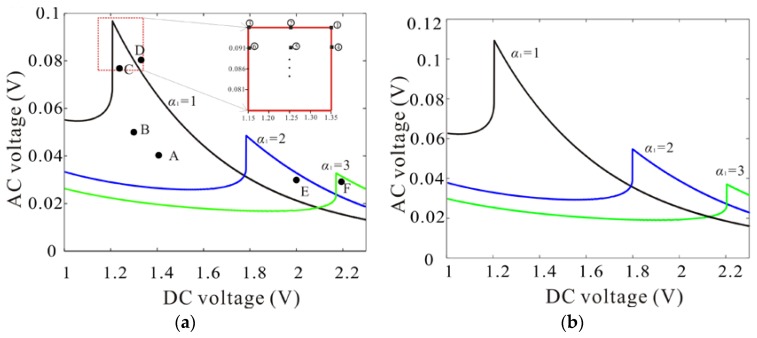
The $V_{ac}-V_{dc}$ parameter space under different ratio of the gap width and the thickness of the mircobeam in the case of η=0.25 (**a**) and η=0.5 (**b**) (The parameter area below the curve is monostable parameter space).

Then, from Equation (35), parameter space of the monostable vibration with the different specification will be studied. As shown in Figure 7, the boundary between monostable vibration and multistable vibration in the case of η=0.25 and η=0.5 is given. When AC voltage is less than that of the boundary, monostable vibration appears. It is found that there is only one solution when the DC voltage or AC voltage is very small, which can be proved with Equation (31). With a small vibration force, the system is equivalent to linear vibration. It is impossible for the system to generate more than one solution. With the increase of vibration force, to obtain the monostable vibration, strict parameter conditions are given. It is noted that there is one peak under the different specification, where the exciting force is put to the maximum. Under the monostable vibration, the system becomes almost linear. And the amplitude of linear vibration is proportional to the exciting force. Then, the maximum amplitude is obtained near the peak in the Figure 7a. What’s more, Figure 7 shows the parameter space of the monostable vibration increases with the increase of the scale effect.

Besides, the AC voltage of the peak decreases as the ratio of the gap width to the mircobeam thickness increases, which can be explained with Figure 5. With the increase of the ratio of the gap width to the thickness of the mircobeam, a relatively large DC voltage is required to counteract hardening of the system. However, as the amplitude increases, the softening is more and more obvious. So, a relatively small AC voltage is needed to prevent bifurcation. On the contrary, if the ratio of the gap width to the mircobeam thickness is too small, a small enough DC voltage is required to counteract hardening. Meanwhile, a relatively large AC voltage is required to produce large amplitude. However, the condition $V_{dc}>>V_{ac}$ is false. The second-order item of the AC voltage cannot be ignored, which can lead to multiple frequency vibration.

The ratio of the gap width to the mircobeam thickness is the key to realize monostable large amplitude vibration and it decides the maximum amplitude that can be realized with appropriate DC and AC voltage. Reasonable decreasing the ratio of the gap width to the mircobeam thickness, the system can realize monostable large amplitude vibration. As we know, monostable large amplitude vibration is desired for many applications, such as microbeam resonator, which can eliminate the dynamic bifurcation phenomenon and increase the vibration energy.

**Figure 8:**
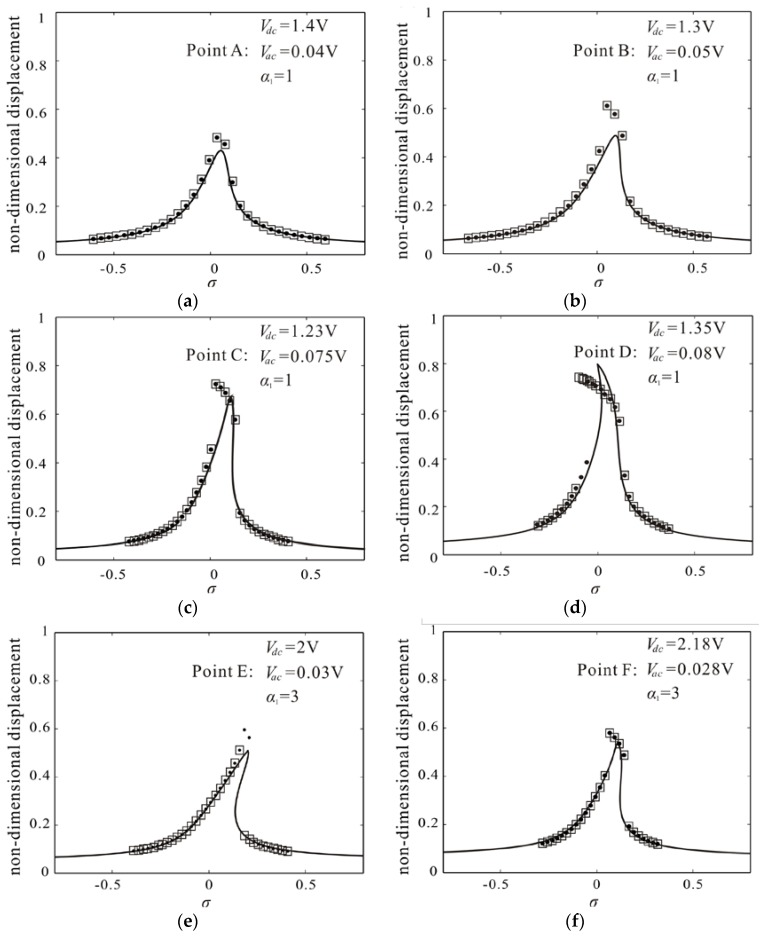
Comparison of the frequency response curve obtained by Nonlinear Galerkin method (solid line) and Differential Quadrature method: (**a**–**f**) corresponding to A–F in Figure 7a (dotted line represents result obtained by sweeping up the frequency; rectangle represents result obtained by sweeping down the frequency).

To verify the validity of the results, the Differential Quadrature method is proposed to compare with the Nonlinear Galerkin method. Figure 8a–f show the frequency response corresponding to A–F shown in Figure 7.

Here, the frequency response is calculated with sweeping up the frequency and sweeping down the frequency by Differential Quadrature method. As shown in Figure 8a–c, f, the results obtained by sweep-up case and sweep-down case are consistent, which conforms to the monostable vibration shown in Figure 7. From Figure 7, the voltage of point D exceeds parameter space of monostable vibration, which leads to spring softening dominance as shown in Figure 8d. Meanwhile, the DC voltage of point E is relatively small, which leads to spring hardening dominance as shown in Figure 8e. From Figure 8, with the increase of the amplitude, the deviation between the results obtained by the Nonlinear Galerkin method and those obtained by Differential Quadrature method becomes more and more obvious. But the error cannot affect our conclusion that the improved single degree of freedom model can study qualitatively monostable large amplitude vibration. To quantitatively study monostable large amplitude vibration, an optimization theory is proposed.

The formula of maximum amplitude $q_{9}V_{ac}/\omega_{1d}c_{nd}$ is used near the peak shown in Figure 7. Then, the maximum amplitude of monostable vibration under different ratio of the gap width to the mircobeam thickness and size effect is predicted. And, an optimization theory, which is based on the following three points, is proposed to verify theoretical prediction results.

(a) Under monostable vibration, $\kappa_{1}$ and $\kappa_{2}$ approximate to zero. The system is equivalent to approximate linear vibration.
(b) The maximum amplitude of approximate linear vibration is proportional to exciting force that is decided with the product of DC voltage and AC voltage.
(c) Optimization parameters, which can realize the maximum amplitude of monostable vibration, are taken near the peak regions in Figure 7 (for example, the red frame with $\alpha_{1}=1$).

**Figure 9:**
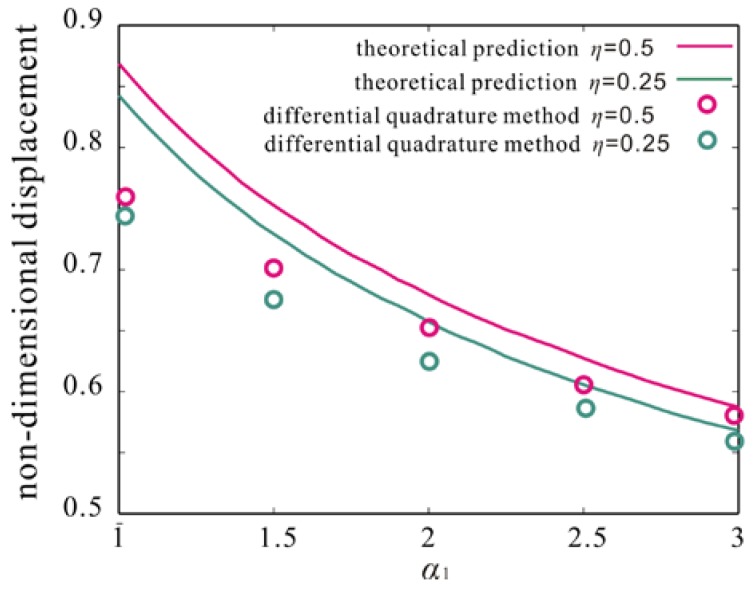
Comparison of the maximum amplitudes obtained by theoretical prediction and Differential Quadrature method.

We take a rectangular area near the peak region and discretize it into many points as shown in Figure 7. As serial number increases, exciting force decreases. Differential Quadrature method is used to calculate the frequency response according to the order. When results obtained by sweeping up the frequency and sweeping down the frequency are consistent, the calculation stops and the maximum amplitude of monostable vibration is obtained as shown in Figure 9. It is found that the maximum amplitude increases with the decrease of the ratio of the gap width to the mircobeam thickness. Meanwhile, as size effect increases, the maximum amplitude increases. The results obtained by theoretical prediction are qualitative agreement with them obtained by Differential Quadrature method. However, theoretical results are larger than numerical ones. With the decrease of the ratio of gap width to thickness of mircobeam, the deviation between theoretical prediction and numerical result becomes more and more obvious. In the next study, in order to improve the calculation accuracy, more space dimensions should be taken in the Nonlinear Galerkin method.

In this section, the improved single degree of freedom model can describe monostable large amplitude vibration qualitatively. Although, when amplitude exceeds half of the gap, the error between reduced-order model and continuum model becomes obvious, our proposed model is significant to study the relationship between maximum amplitude and physical parameter.

## 5. Conclusions

With the Nonlinear Galerkin method, we propose a novel approach to generate an improved single degree of freedom model for electrically actuated microbeam-based MEMS and use it to study the static and dynamic behaviors of these devices. Specially, the monostable vibration is theoretically investigated in size effected MEMS via the low dimensional model. The proposed theoretical results maintain a good situation consistency with the results obtained by Differential Quadrature method. Besides, the Finite element results of case studies are used to verify the accuracy of the model. Compared with the results obtained by the Linear Galerkin method, the model has obvious superiority.

What’s more, the monostable large amplitude vibration eliminates dynamic bifurcation phenomenon, improves the stability of the system and increases the vibration energy, which is desired for many applications. Parameter space and maximum amplitude of the monostable vibration are obtained by using unfolding analysis and optimization theory for the first time. It is found that reasonable decreasing the ratio of the gap width to the thickness of the mircobeam is the key to realize monostable large amplitude vibration. Besides, the Nonlinear Galerkin method gives a way to convert partial differential equation into ordinary differential equation.

## Figures and Tables

**Table 1:** Part of design parameters for a microbeam-based resonator.

Parameter	Value	Units
Mass density *ρ*	2300	kg/m^3^
Young’s modulus *E*	169	Gpa
Beam length *L*	365	μm
Beam width *b*	10	μm
Beam thickness *h*	1	μm
Axial load $\hat{N}$	variable	N
Viscous damping *c*	3.42 × 10^−5^	Ns/m^2^

**Table 2:** Natural frequency under different DC voltage when d=1 μm .

Case	DC Voltage (V)	Linear Galerkin Method Results (kHz)	Nonlinear Galerkin Method Results (kHz)	Finite Element Results (kHz)	Error
1	2	62.44	62.77	62.82	0.6%; 0.1%
2	2.5	59.85	60.42	60.54	1.1%; 0.2%
3	3	55.53	56.55	56.87	2.4%; 0.6%
4	3.5	44.33	47.59	48.71	9.0%; 2.3%

**Table 3:** Pull-in voltages under different gap width.

Case	Gap Width (μm)	Linear Galerkin Method Results (V)	Nonlinear Galerkin Method Results (V)	Finite Element Results (V)	Error
1	0.5	1.25	1.27	1.27	1.6%; 0%
2	1	3.66	3.74	3.76	2.7%; 0.5%
3	1.5	7.16	7.39	7.50	4.5%; 1.5%
4	2	11.94	12.47	12.85	7.1%; 3.0%

## Appendix A

Equations (A1)–(A15) represent the coefficients produced in the Nonlinear Galerkin process; Equations (A16)–(A25) represent the coefficients that are obtained by eliminating static displacement; Equations (A26)–(A29) represent the coefficients produced in the perturbation process.

(A1) $$n_{0}=1-2\frac{\alpha_{2}V_{dc}^{2}\int_{0}^{1}\phi_{1}^{2}\phi_{3}dx\int_{0}^{1}\phi_{3}dx}{\omega_{3}^{2}}$$

(A2) $$n_{1}=-2\int_{0}^{1}\phi_{1}^{3}dx+2\frac{\alpha_{2}V_{dc}^{2}}{\omega_{3}^{2}}\int_{0}^{1}\phi_{1}^{3}\phi_{3}dx\int_{0}^{1}\phi_{3}dx$$

(A3) $$n_{2}=\int_{0}^{1}\phi_{1}^{4}dx-4{\left(\int_{0}^{1}\phi_{1}^{2}\phi_{3}dx\right)}^{2}\frac{\omega_{1}^{2}}{\omega_{3}^{2}}$$

(A4) $$n_{3}=6\frac{\omega_{1}^{2}}{\omega_{3}^{2}}\int_{0}^{1}\phi_{1}^{2}\phi_{3}dx\int_{0}^{1}\phi_{1}^{3}\phi_{3}dx-\frac{2\alpha_{1}}{\omega_{3}^{2}}\int_{0}^{1}\phi_{1}^{2}\phi_{3}dx\int_{0}^{1}{\phi_{1}}^{\prime \prime }\phi_{3}dx\int_{0}^{1}{\phi_{1}^{\prime }}^{2}dx$$

(A5) $$n_{4}=\frac{4\alpha_{1}}{\omega_{3}^{2}}\int_{0}^{1}\phi_{1}^{2}\phi_{3}dx\int_{0}^{1}\phi_{1}{\phi_{1}}^{\prime \prime }\phi_{3}dx\int_{0}^{1}{\phi_{1}^{\prime }}^{2}dx-2\frac{\omega_{1}^{2}}{\omega_{3}^{2}}{\left(\int_{0}^{1}\phi_{1}^{3}\phi_{3}dx\right)}^{2}+\frac{2\alpha_{1}}{\omega_{3}^{2}}\int_{0}^{1}\phi_{1}^{3}\phi_{3}dx\int_{0}^{1}{\phi_{1}}^{\prime \prime }\phi_{3}dx\int_{0}^{1}{\phi_{1}^{\prime }}^{2}dx$$

(A6) $$m_{1}=\alpha_{1}\int_{0}^{1}{\phi_{1}}^{\prime \prime }\phi_{1}dx\int_{0}^{1}{\phi_{1}^{\prime }}^{2}dx+4\omega_{3}^{2}{\left(\int_{0}^{1}\phi_{1}^{2}\phi_{3}dx\right)}^{2}\frac{\omega_{1}^{2}}{\omega_{3}^{2}}$$

(A7) $$m_{2}=4{\left(\int_{0}^{1}\phi_{1}^{2}\phi_{3}dx\right)}^{2}$$

(A8) $$m_{3}=2\alpha_{2}V_{dc}^{2}\int_{0}^{1}\phi_{1}^{2}\phi_{3}dx\int_{0}^{1}\phi_{3}dx$$

(A9) $$m_{4}=\alpha_{2}V_{dc}^{2}\int_{0}^{1}\phi_{1}dx$$

(A10) $$m_{5}=\alpha_{2}\int_{0}^{1}\phi_{1}dx$$

(A11) $$\chi =-\omega_{3}^{2}\int_{0}^{1}\phi_{1}^{3}\phi_{3}dx+2\alpha_{1}\int_{0}^{1}{\phi_{1}}^{\prime \prime }\phi_{1}dx\int_{0}^{1}\phi_{1}^{\prime }\phi_{3}^{\prime }dx+\alpha_{1}\int_{0}^{1}\phi_{1}^{2}dx\int_{0}^{1}\phi_{1}\phi_{3}^{\prime \prime }dx$$

(A12) $$m_{6}=\chi \frac{\alpha_{2}V_{dc}^{2}}{\omega_{3}^{2}}\int_{0}^{1}\phi_{3}dx$$

(A13) $$m_{7}=2\omega_{3}^{2}\int_{0}^{1}\phi_{1}^{2}\phi_{3}dx(\frac{\alpha_{1}}{\omega_{3}^{2}}\int_{0}^{1}{\phi_{1}}^{\prime \prime }\phi_{3}dx\int_{0}^{1}{\phi_{1}^{\prime }}^{2}dx-\frac{\omega_{1}^{2}}{\omega_{3}^{2}}\int_{0}^{1}\phi_{1}^{3}\phi_{3}dx)+\frac{2\omega_{1}^{2}\chi }{\omega_{3}^{2}}\int_{0}^{1}\phi_{1}^{2}\phi_{3}dx-2\alpha_{1}\int_{0}^{1}{\phi_{1}^{\prime }}^{2}dx\int_{0}^{1}\phi_{1}^{2}{\phi_{1}}^{\prime \prime }dx$$

(A14) $$m_{8}=-2\int_{0}^{1}\phi_{1}^{2}\phi_{3}dx\int_{0}^{1}\phi_{1}^{3}\phi_{3}dx+\frac{2\chi }{\omega_{3}^{2}}\int_{0}^{1}\phi_{1}^{2}\phi_{3}dx$$

(A15) $$m_{9}=\alpha_{1}\int_{0}^{1}{\phi_{1}^{\prime }}^{2}dx\int_{0}^{1}\phi_{1}^{3}{\phi_{1}}^{\prime \prime }dx-4\alpha_{1}\int_{0}^{1}\phi_{1}^{2}\phi_{3}dx\int_{0}^{1}\phi_{1}{\phi_{1}}^{\prime \prime }\phi_{3}dx\int_{0}^{1}{\phi_{1}^{\prime }}^{2}dx+\frac{\alpha_{1}\chi }{\omega_{3}^{2}}\int_{0}^{1}\phi_{1}^{2}dx\int_{0}^{1}\phi_{1}{\phi^{\prime \prime }}_{3}dx-\frac{\omega_{1}^{2}\chi }{\omega_{3}^{2}}\int_{0}^{1}\phi_{1}^{3}\phi_{3}dx$$

(A16) $$q_{1}=\frac{-(n_{1}+2n_{2}u_{1s}+3n_{3}u_{1s}^{2}+4n_{4}u_{1s}^{3})+2m_{2}u_{1s}+3m_{8}u_{1s}^{2}}{n_{0}+n_{1}u_{1s}+(n_{2}-m_{2})u_{1s}^{2}+(n_{3}-m_{8})u_{1s}^{3}+n_{4}u_{1s}^{4}}$$

(A17) $$q_{2}=\frac{m_{2}-n_{2}+3(m_{8}-n_{3})u_{1s}-6n_{4}u_{1s}^{2}}{n_{0}+n_{1}u_{1s}+(n_{2}-m_{2})u_{1s}^{2}+(n_{3}-m_{8})u_{1s}^{3}+n_{4}u_{1s}^{4}}$$

(A18) $$q_{3}=\frac{m_{8}-n_{3}-4n_{4}u_{1s}}{n_{0}+n_{1}u_{1s}+(n_{2}-m_{2})u_{1s}^{2}+(n_{3}-m_{8})u_{1s}^{3}+n_{4}u_{1s}^{4}}$$

(A19) $$q_{4}=\frac{-n_{4}}{n_{0}+n_{1}u_{1s}+(n_{2}-m_{2})u_{1s}^{2}+(n_{3}-m_{8})u_{1s}^{3}+n_{4}u_{1s}^{4}}$$

(A20) $$q_{5}=\frac{-\omega_{1}^{2}(3n_{2}u_{1s}+n_{1}+6n_{3}u_{1s}^{2}+10n_{4}u_{1s}^{3})+m_{6}+3m_{1}u_{1s}+6m_{7}u_{1s}^{2}+10m_{9}u_{1s}^{3}}{n_{0}+n_{1}u_{1s}+(n_{2}-m_{2})u_{1s}^{2}+(n_{3}-m_{8})u_{1s}^{3}+n_{4}u_{1s}^{4}}$$

(A21) $$q_{6}=\frac{-\omega_{1}^{2}(n_{2}+4n_{3}u_{1s}+10n_{4}u_{1s}^{2})+m_{1}+4m_{7}u_{1s}+10m_{9}u_{1s}^{2}}{n_{0}+n_{1}u_{1s}+(n_{2}-m_{2})u_{1s}^{2}+(n_{3}-m_{8})u_{1s}^{3}+n_{4}u_{1s}^{4}}$$

(A22) $$q_{7}=\frac{-\omega_{1}^{2}(n_{3}+5n_{4}u_{1s})+m_{7}+5m_{9}u_{1s}}{n_{0}+n_{1}u_{1s}+(n_{2}-m_{2})u_{1s}^{2}+(n_{3}-m_{8})u_{1s}^{3}+n_{4}u_{1s}^{4}}$$

(A23) $$q_{8}=\frac{m_{9}-\omega_{1}^{2}n_{4}}{n_{0}+n_{1}u_{1s}+(n_{2}-m_{2})u_{1s}^{2}+(n_{3}-m_{8})u_{1s}^{3}+n_{4}u_{1s}^{4}}$$

(A24) $$q_{9}=\frac{2m_{5}V_{dc}}{n_{0}+n_{1}u_{1s}+(n_{2}-m_{2})u_{1s}^{2}+(n_{3}-m_{8})u_{1s}^{3}+n_{4}u_{1s}^{4}}$$

(A25) $$\omega_{1d}^{2}=\frac{(n_{0}+2n_{1}u_{1s}+3n_{2}u_{1s}^{2}+4n_{3}u_{1s}^{3}+5n_{4}u_{1s}^{4})\omega_{1}^{2}-5m_{9}u_{1s}^{4}-4m_{7}u_{1s}^{3}-3m_{1}u_{1s}^{2}-2m_{6}u_{1s}-m_{3}}{n_{0}+n_{1}u_{1s}+(n_{2}-m_{2})u_{1s}^{2}+(n_{3}-m_{8})u_{1s}^{3}+n_{4}u_{1s}^{4}}$$

(A26) $$\chi_{1}=q_{2}\omega_{1d}^{2}-q_{6}+\frac{q_{1}\omega_{1d}^{2}-q_{5}}{3\omega_{1d}^{2}}(5q_{1}\omega_{1d}^{2}-2q_{5})$$

(A27) $$\chi_{2}=\frac{q_{1}\omega_{1d}^{2}-q_{5}}{\omega_{1d}^{2}}[q_{6}+2q_{2}\omega_{1d}^{2}+\frac{4}{3}\kappa_{1}+\frac{q_{1}\omega_{1d}^{2}-q_{5}}{3\omega_{1d}^{2}}(4q_{5}-8q_{1})]-4q_{7}+4q_{3}\omega^{2}-q_{1}\kappa_{1}+\frac{9q_{1}\omega_{1d}^{2}-2q_{5}}{8\omega_{1d}^{2}}\chi_{1}$$

(A28) $$\chi_{3}=\frac{q_{1}\omega_{1d}^{2}-q_{5}}{9\omega_{1d}^{2}}(19q_{5}-4q_{1}\omega_{1d}^{2}-45q_{6}+6q_{2}\omega_{1d}^{2})+3q_{7}-3q_{3}\omega_{1d}^{2}+q_{1}\kappa_{1}$$

(A29) $$\begin{array}{ll} \kappa_{2} & =(\frac{\chi_{2}}{3\omega_{1d}^{2}}+\frac{2\chi_{3}}{\omega_{1d}^{2}})(2q_{5}-5q_{1}\omega_{1d}^{2})+\chi_{1}[\frac{3q_{6}-11q_{2}\omega_{1d}^{2}}{8\omega_{1d}^{2}}+\frac{q_{1}\omega_{1d}^{2}-q_{5}}{\omega_{1d}^{2}}(\frac{q_{5}}{12\omega_{1d}^{2}}-\frac{q_{1}}{2})]\\ & +\frac{q_{1}\omega_{1d}^{2}-q_{5}}{\omega_{1d}^{2}}[\frac{q_{1}\omega_{1d}^{2}-q_{5}}{\omega_{1d}^{2}}(\frac{16q_{6}}{3}-\frac{8q_{2}\omega_{1d}^{2}}{3})-\frac{56}{3}q_{7}+\frac{26}{3}q_{3}\omega_{1d}^{2}]+\kappa_{1}(\frac{q_{5}-q_{1}\omega_{1d}^{2}}{3\omega_{1d}^{2}}q_{1}+3q_{2})\\ & +10q_{8}-10\omega_{1d}^{2}q_{4} \end{array}$$
